# The role of seasonal flowering responses in adaptation of grasses to temperate climates

**DOI:** 10.3389/fpls.2014.00431

**Published:** 2014-08-29

**Authors:** Siri Fjellheim, Scott Boden, Ben Trevaskis

**Affiliations:** ^1^Department of Plant Sciences, Norwegian University of Life Sciences, ÅsNorway; ^2^Division of Plant Industry, Commonwealth Scientific and Industrial Research Organisation, Canberra, ACTAustralia

**Keywords:** flowering, adaptation, biological, evolution, molecular, seasonality, Pooideae

## Abstract

Grasses of the subfamily Pooideae, including important cereal crops and pasture grasses, are widespread in temperate zones. Seasonal regulation of developmental transitions coordinates the life cycles of Pooideae with the passing seasons so that flowering and seed production coincide with favorable conditions in spring. This review examines the molecular pathways that control the seasonal flowering responses of Pooideae and how variation in the activity of genes controlling these pathways can adapt cereals or grasses to different climates and geographical regions. The possible evolutionary origins of the seasonal flowering responses of the Pooideae are discussed and key questions for future research highlighted. These include the need to develop a better understanding of the molecular basis for seasonal flowering in perennial Pooideae and in temperate grasses outside the core Pooideae group.

## OVERVIEW

Grasses from the Pooideae subfamily, family Poaceae, occur widely in temperate regions (Hartley, 1973). In addition to being important ecologically this group includes economically important pasture grasses and cereal crops, as well as invasive species and weeds. Adaptations that allow Pooid grasses to survive the seasonal extremes of temperate climates are of interest since these are central to the success of this sub-family across temperate zones. Such adaptations are also important to agriculture and are critical to the success of temperate cereal crops including wheat, barley, oats, and rye.

A key factor underlying the adaptation of grasses to temperate climates is timing. Timing biological events to occur during specific seasons is the basis for stress avoidance strategies. For example, delaying flowering until after winter allows many grasses to avoid frost damage to cold sensitive reproductive organs (see Fowler et al., 1996). Alternatively, activation of tolerance mechanisms at particular times of year can prime plant physiology for predictably harsh seasonal conditions before these occur. An example of seasonally timed tolerance is the cold acclimation response, where freezing tolerance increases during autumn to maximize chances of winter survival (Thomashow, 2010).

The study of the seasonal timing of biological phenomena is known as phenology. The broad topic of this review is the contribution of phenology to the success of Pooideae in temperate climates. We will highlight how different seasonal cues can adjust the developmental program of grasses to match seasonal cycles, using temperate cereals as examples, and then examine how naturally occurring variation in these responses drives adaptation to different geographical regions and climates. Then we will discuss how the molecular pathways controlling the seasonal flowering responses of temperate grasses might have evolved.

## DEVELOPMENTAL PHASES OF TEMPERATE CEREALS AND GRASSES

**FIGURE 1:**
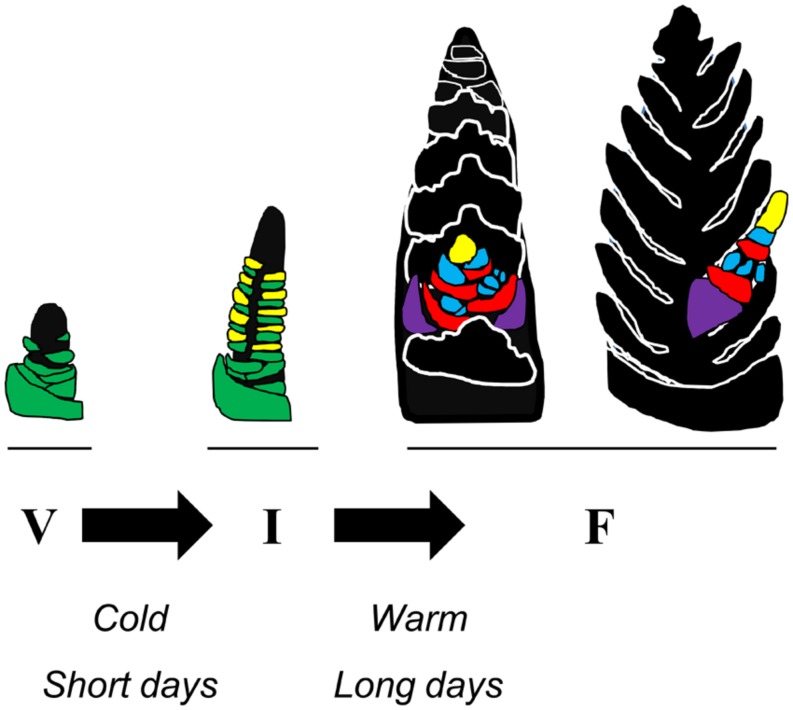
**Schematic representation of how seasonal conditions influence the developmental transitions of wheat.** At the beginning of the crop life cycle there is a vegetative meristem at the tip of the shoot apex of the main tiller. Leaf primordia (green) develop below the meristem. During winter short days or prolonged cold can promote the transition to reproductive development. This allows inflorescence initiation (I) to occur. The shoot apex elongates and inflorescence primordia (yellow) appear above each of the leaf primordia. During subsequent stages of inflorescence development the lateral inflorescence meristems develop into branches (the spikelets) upon which florets develop, whereas development of leaf primordia ceases. During spring long days and high temperatures can accelerate inflorescence development. Two orientations of the developing inflorescence are represented (F, floral development) with a single spikelet highlighted and the rest of the spike shown in silhouette. Each spikelet has 2 glumes (purple) and then a series of florets at an early stage of development. The lemma primordia (red) and floral organ primordia (anther primordia, blue) are shown, along with the lateral inflorescence meristem at the end of the spikelet (yellow). Each unit of lemma and floral organ primordia will differentiate into a floret.

The developmental phases of temperate cereals and related temperate grasses can be divided into two broad stages: vegetative and reproductive. During the vegetative phase the shoot apex produces only leaf primordia. Then, when the transition to reproductive growth occurs, inflorescence primordia develop above leaf primordia at the apex. This stage is referred to as inflorescence initiation (Evans, 1964). Subsequently the inflorescence primordia develop into lateral inflorescence branches, called spikelets, whereas the development of the leaf primordia ceases (**Figure 1**). As inflorescence development proceeds floral primordia appear on the developing spikelets and these develop into flowers, referred to as florets (see Barnard, 1964). Various arrangements of spikelet number, branch length, and floret number exist, giving rise to diverse inflorescence shapes, from compact spikes (e.g., wheat and barley) to panicles (e.g., oats). Internodes beneath the developing inflorescence elongate to form the stem, carrying the inflorescence upwards inside a sheath of leaves. The inflorescence emerges from the leaf sheath shortly before anthesis. In annual grasses the transition from vegetative to reproductive development occurs once. The situation for perennial grasses is more complex and will be discussed later.

## SEASONAL REGULATION OF DEVELOPMENT PHASES IN TEMPERATE GRASSES

The different developmental phases of temperate grasses (limited to Pooid grasses in this review) are timed to optimally coordinate the plant life cycle with the changing seasons. This timing is achieved through the influence of seasonal changes in temperature and daylength on specific points in the developmental program (**Figure 1**). Initially there is a delay of inflorescence initiation prior to winter. This delay is removed when plants over-winter, allowing inflorescence initiation to occur. Subsequently, increasing daylength and warmer temperatures accelerate development of the inflorescence in spring (Purvis, 1934; Evans, 1964). The overall combination of these seasonal developmental responses ensures that plants remain vegetative before winter, avoiding frost damage to developing reproductive organs, but then flower rapidly in spring, to allow flowering and seed production before the onset of heat and water limitation in summer.

## THE RELATIONSHIP BETWEEN DEVELOPMENTAL PHASES AND OTHER TRAITS

Seasonal timing of developmental phases influences other traits. Sometimes this is simply a matter of timing. For example, if there is limited water at the end of the growing season the timing of flowering (heading date) will influence grain yield of cereal crops because early flowering lines can produce grain before water becomes limiting whereas late flowering lines encounter drought stress, which reduces yield. There are many field studies that demonstrate indirect effects caused by the timing of flowering (Kuchel et al., 2007, for example). In other instances there is a direct relationship between development and other traits. The best understood example is the relationship between flowering and frost tolerance in wheat and barley. During the vegetative growth phase wheat and barley plants can acclimate to cold if exposed to non-freezing low temperatures for a period of weeks and will subsequently survive extreme winter conditions. This capacity to acclimate to cold decreases as plants develop toward flowering (Fowler et al., 2001; Limin and Fowler, 2006). Thus, seasonal regulation of development coordinates the potential for cold acclimation with seasonal conditions; vegetative plants can acclimate to cold during autumn and winter, but this capacity is lost as plants begin to flower in spring, when there is less risk of frost. The influence of developmental phases on the seasonal activation of abiotic and biotic stress tolerances is an important component of the adaptation of grasses to temperate climates.

## MOLECULAR PATHWAYS CONTROLLING SEASONAL FLOWERING RESPONSES IN *Arabidopsis*

*Arabidopsis* (*Arabidopsis thaliana*) is adapted to temperate climates and, like temperate grasses, flowers in response to vernalization (the prolonged cold of winter) and long days (see Amasino, 2010). *Arabidopsis* provides a useful guide to the types of genes/proteins that regulate flowering and, although there are important differences, the molecular mechanisms controlling seasonal flowering responses of grasses can be viewed as variations of those that also occur in *Arabidopsis*.

Seasonal flowering of *Arabidopsis* is controlled through regulation of the floral transition, the point when the floral primordia appear at the shoot apex and flowering begins. In winter annual *Arabidopsis* ecotypes the floral transition is delayed until plants experience vernalization. This delay is caused by *FLOWERING LOCUS C* (*FLC*), which encodes a MADS box transcription factor that represses flowering (Michaels and Amasino, 1999; Sheldon et al., 1999). *FLC* is expressed at high basal levels prior to vernalization (Michaels and Amasino, 1999; Sheldon et al., 1999). Prolonged exposure to cold represses transcription of *FLC*, through mechanisms that act at the chromatin of the *FLC* gene to limit transcription. For example, tri-methylation of histone-3-lysine 27 (H3K27Me3) in nucleosomes at the *FLC* locus is required to maintain repression of *FLC* in vernalized plants (Bastow et al., 2004; Schubert et al., 2006; Finnegan and Dennis, 2007). Stable repression of *FLC* after vernalization provides a molecular memory of winter. Genetic variation in *FLC* activity, either due to mutations in the *FLC* gene itself or in regulators of *FLC* expression, can reduce or eliminate the ancestral vernalization requirement of *Arabidopsis* and adapt ecotypes to different climates (see Amasino, 2010).

Long days accelerate the floral transition of *Arabidopsis*. The long-day flowering response depends on an internal timekeeping mechanism, the circadian clock, which activates expression of *CONSTANS* (*CO*) in the late afternoon (Suarez-Lopez et al., 2001). The CO protein is stable in light (Valverde et al., 2004). Thus, when days are long *CO* expression coincides with light and the protein is active. This activates transcription of *FLOWERING LOCUS T* (*FT*) in the leaves. *FT* encodes a phosphatidyl-ethanolamine binding protein (PEBP) that is translocated through the phloem from the leaves to the shoot apex (Kardailsky et al., 1999; Kobayashi et al., 1999; Corbesier et al., 2007). At the shoot apex the FT protein interacts with *FLOWERING LOCUS D*, a bZIP transcription factor (Abe et al., 2005; Wigge et al., 2005). The resulting complex activates transcription of genes that promote floral development, including *AP1*, thus triggering the floral transition. Activation of *FT* by CO is likely to be mediated through interactions with nuclear factor Y proteins (NF-Y) – also known as heme activator proteins (HAP) or CCAAT binding factors (referred to hereafter as NF-Y proteins; Ben-Naim et al., 2006; Wenkel et al., 2006). These are components of a conserved eukaryotic transcriptional activation complex. Interactions between CO and NF-Y proteins are mediated by the CCT domain; a protein domain first identified in CO, CO-like and TIMING OF CAB1 EXPRESSION1 proteins (Wenkel et al., 2006). Long-day activation of *FT* is suppressed in *Arabidopsis* plants that have not been vernalized (Lee et al., 2000; Michaels et al., 2005). This is due to direct repression of by FLC, which binds to the intron of the *FT* gene (Helliwell et al., 2006).

High temperatures (>25°C) can activate expression of *FT* in short days and accelerate the floral transition (Blázquez et al., 2003; Balasubramanian et al., 2006; Turck et al., 2008). Elevated temperatures during the night activate *FT* expression. Activation of *FT* by high-temperatures is largely independent of *CO* and instead *PHYTOCHROME-INTERACTING FACTORS* (*PIF4* and *PIF5*) are required (Kumar et al., 2012; Thines et al., 2014). The *FLC*-like gene *FLOWERING LOCUS M*1* (FLM*1*)* has also been implicated in the regulation of the high-temperature flowering response of *Arabidopsis* (Balasubramanian et al., 2006). This gene is differentially spliced in a temperature dependent manner, producing a splice variant that represses flowering at lower temperatures (Lee et al., 2013; Posé et al., 2013).

As outlined in the next section, many of the classes of genes/proteins that regulate seasonal flowering responses in *Arabidopsis* also play important roles in regulating reproductive development in cereals. There are, however, few direct overlaps between *Arabidopsis* and cereals, with fundamental differences in the physiology of seasonal flowering responses and in the underlying molecular networks.

## MOLECULAR NETWORKS CONTROLLING SEASONAL FLOWERING RESPONSES IN TEMPERATE CEREALS

There exists considerable diversity in seasonal flowering responses amongst temperate cereals. Some lack the requirement for vernalization, others are daylength insensitive. For the purpose of this review we will consider the presence of a vernalization requirement combined with daylength sensitivity as being the archetypal flowering behavior of temperate cereals. This view is supported by molecular evidence (see subsequent sections) that reduced vernalization requirement and reduced daylength sensitivity are acquired states that have arisen on multiple occasions through mutation.

### THE VERNALIZATION REQUIREMENT DELAYS INFLORESCENCE INITIATION BEFORE WINTER

A core feature of the flowering behavior of temperate cereals and related grasses is the delay of inflorescence initiation before winter; the vernalization requirement. For example, many wheats and barleys will grow vegetatively for extended periods and will not flower without overwintering. Genetic studies of wheat and barley have identified the *VRN2* locus as a key gene controlling vernalization requirement in the cereals (Takahashi and Yasuda, 1971). Duplicated zinc finger-CCT domain proteins are found at the *VRN2* locus (Yan et al., 2004a). Some accessions of barley and diploid wheats lack a functional copy of *VRN2* due to loss-of-function mutations in the *VRN2* coding sequence or due to naturally occurring deletions of the entire *VRN2* locus (Yan et al., 2004a; Dubcovsky et al., 2005). These accessions can undergo rapid inflorescence initiation without vernalization. This only occurs in long days, however, where rapid flowering is associated with elevated expression *FT-like 1* (*FT1*), the functional equivalent of *FT* in cereals (Karsai et al., 2005; Turner et al., 2005; Hemming et al., 2008). This suggests that the normal role for *VRN2* is to block long-day induction of *FT1*. Consistent with this hypothesis, transcription of *VRN2* occurs in long days (12 h light or longer) and constitutive expression of *VRN2* down-regulates *FT1* (Trevaskis et al., 2006; Hemming et al., 2008). The VRN2 protein is distantly related to CO and, like CO, can interact with NF-Y proteins via a CCT domain (Li et al., 2011). Binding of VRN2 to NF-Y proteins might inhibit transcription of *FT1* by blocking binding of other CCT domain proteins that normally activate transcription of *FT1*, such as the cereal CO homologs.

### SHORT DAYS ALLOW INFLORESCENCE INITIATION

Cereal varieties that are unable to flower without vernalization when grown in long days can flower in short days, suggesting that inhibition of inflorescence initiation is weaker in short days (Evans, 1987). This is also evidenced by “short-day vernalization,” where plants grown in short days for several weeks will flower when shifted to long-days (Purvis and Gregory, 1937). Low-levels of *VRN2* expression in short-days might allow inflorescence initiation in short-days (Dubcovsky et al., 2006; Turner et al., 2013). This is unlikely to occur through *FT1*, which is expressed mainly in long days. Another *FT*-like gene (*FT3*) might be important. *FT3* is normally expressed in short but not long days, and loss-of-function mutations in *FT3* delay flowering primarily in short days (Faure et al., 2007; Kikuchi et al., 2009). Interestingly, *FT3* is expressed at high levels in long days in lines that lack *VRN2*. So daylength specificity of *FT3* expression might be mediated by *VRN2* (Casao et al., 2011). It is not known whether loss-of-function mutations in *FT3* block the short-day vernalization effect. The mechanisms controlling inflorescence initiation in short-days are an interesting area for future research.

### THE PROLONGED COLD OF WINTER PROMOTES INFLORESCENCE INITIATION

Prolonged exposure to low-temperatures (vernalization) promotes inflorescence initiation (Chouard, 1960). Vernalization can be applied to imbibed seeds or to actively growing plants and is effective irrespective of daylength, even in total darkness (Gassner, 1918; Gott et al., 1955). The effect of vernalization on plant development can be separated from the actual low-temperature treatment. For example, vernalization of imbibed seeds promotes rapid inflorescence initiation when plants are subsequently shifted to normal glasshouse conditions (Sasani et al., 2009, for example). Thus, there is a memory of prolonged cold treatment. Typically temperatures between 0 and 10°C are effective for vernalization and the effect of cold is quantitative, with longer cold treatments causing more rapid inflorescence initiation until a point when further cold causes no further reduction in the time taken to flower; the vernalization saturation point (Gassner, 1918; Gott et al., 1955).

The promotion of inflorescence initiation by vernalization is stronger than the effect of short days. For example, in experiments performed by Allard et al. (2012) wheat plants exposed to prolonged low-temperatures (5°C for 30 days) flowered with 8 leaves (primary tiller). Typically there are four to five leaf primordia present at the time of germination and these develop into leaves irrespective of conditions during subsequent growth, so a final leaf number of 8 is indicative of a rapid progression toward flowering. In comparison, plants grown without vernalization in long days flowered with 16 leaves. Plants grown in short days for 3–10 weeks then shifted to long days flowered with 12 leaves, showing that short days can promote inflorescence initiation, though the effect is weaker than that of prolonged cold (12 leaves following short day treatment versus 8 leaves after vernalization). At a broader level the effect of prolonged cold (vernalization *sensu stricto*) is profoundly different to that of short-day treatment because vernalization at low temperatures slows growth. This is particularly evident with vernalization of imbibed seeds where plants can emerge from prolonged cold treatment with only one or two expanded leaves. In comparison plants exposed to several weeks of short days will have many leaves on the main stem and also secondary tillers. Thus, the term “short-day vernalization” should be interpreted with caution, as noted by Evans (1987) who preferred the term “short-day induction.”

The central gene controlling vernalization in cereals is *VERNALIZATION1* (*VRN1*), a MADS box transcription factor gene related to the *AP1/FRUITFULL* genes of *Arabidopsis* (Danyluk et al., 2003; Trevaskis et al., 2003; Yan et al., 2003; Trevaskis, 2010). *VRN1* promotes inflorescence initiation but is expressed at low levels prior to vernalization and this limits the rate of progression toward inflorescence initiation (Danyluk et al., 2003; Murai et al., 2003; Trevaskis et al., 2003; Yan et al., 2003). Exposure to low temperatures induces transcription of *VRN1* (Danyluk et al., 2003; Murai et al., 2003; Trevaskis et al., 2003; Yan et al., 2003). This begins rapidly with the onset of cold, within 12 h, but initial expression is weak and several weeks of cold are required to elevate *VRN1* transcript levels to a level that promotes rapid inflorescence initiation (Sasani et al., 2009; Oliver et al., 2013). There is a strong relationship between the length of cold experienced, *VRN1* expression levels, and the degree to which inflorescence initiation is accelerated post-vernalization (Sasani et al., 2009). These observations are all consistent with a model where stable induction of *VRN1* provides a quantitative memory of vernalization (Trevaskis et al., 2007; Trevaskis, 2010). Indeed, transcriptome analyses show that *VRN1* is one of a limited number of genes that show lasting changes in expression levels in response to seed vernalization (Greenup et al., 2011). Low temperatures induce changes in histone modification of nucleosomes associated with *VRN1*, which might provide a mechanism for stable activation of this gene (Oliver et al., 2009, 2013).

Mutations that disrupt *VRN1* function greatly reduce the impact of vernalization, supporting a central role for *VRN1* in the vernalization response (Chen and Dubcovsky, 2012). Conversely, naturally occurring mutations in the promoter or large insertions/deletions in the first intron of *VRN1* are associated with elevated basal transcription and cause rapid flowering without vernalization (Yan et al., 2004b; Fu et al., 2005). These mutations have been used to breed and select cereal cultivars with reduced vernalization requirement, which are grown in regions or at times of year when vernalization does not occur (see below).

### LONG DAYS ACCELERATE INFLORESCENCE DEVELOPMENT AFTER VERNALIZATION

Daylength can influence both the timing of inflorescence initiation and the rate of inflorescence development after vernalization. In long days vernalized plants will progress rapidly through inflorescence initiation and subsequent stages of inflorescence development until head emergence and anthesis. In short days inflorescence initiation occurs, though not as rapidly as in long days, and thereafter inflorescence development occurs slowly and there is a strong delay of head emergence and anthesis (e.g., Limin and Fowler, 2006; Sasani et al., 2009).

Although long days can accelerate inflorescence initiation in glasshouse experiments this is unlikely to occur in the field for autumn sown varieties (vernalization requiring cultivars, see comments above regarding archetypal flowering behavior), which typically undergo inflorescence initiation in short days toward the end of winter. Instead longer days are likely to coincide with the inflorescence development and stem elongation stage (**Figure 1**). Daylength shift experiments, where wheat plants are shifted from short to long days, or *vice versa*, at different time points, show that the time from inflorescence initiation to the point when the terminal spikelet develops is a critical window for the acceleration of inflorescence development by long daylengths (Miralles and Richards, 2000).

Key genes controlling the long-day flowering response are *PHOTOPERIOD1* (*PPD1*) and *FT*, the cereal *FT* equivalent (Turner et al., 2005; Shaw et al., 2012). Expression of *FT1* is induced by long days, analogous to *FT* in *Arabidopsis*, and it seems that the role for the FT protein as a leaf expressed mobile flowering signal is conserved in cereals (Turner et al., 2005; Tamaki et al., 2007). Long-day induction of *FT1* requires *PPD1*. *PPD1* encodes a pseudo response regulator related to components of the circadian clock and includes a CCT domain that can interact with NF-Y complexes, similar to CO (Turner et al., 2005; Li et al., 2011). Transcription of *PPD1* follows a distinctive diurnal profile, though current models suggest that *PPD1* does not play a role in circadian clock *per se*, and instead regulates outputs of the clock under long days to induce expression of *FT1* (Turner et al., 2005; Campoli et al., 2012).

Deletions in the promoter of the wheat *PPD1* gene are associated with elevated transcript levels of this gene at night, in short daylengths (Beales et al., 2007). These deletions are linked to elevated *FT1* expression and reduced long-day requirement (photoperiod insensitivity), such that plants flower early irrespective of daylength (Beales et al., 2007). Mutations that alter circadian clock rhythms can also up regulate *FT1* and reduce photoperiod sensitivity, loss of *EARLY FLOWERING 3* (*ELF3*, *MAT.a8*) or *PHYTOCLOCK1/LUX1* function, for example (Faure et al., 2012; Mizuno et al., 2012; Zakhrabekova et al., 2012; Campoli et al., 2013; Gawroñski et al., 2014). Genetic activation of the *FT1* gene itself reduces daylength sensitivity, triggering constitutive early flowering (Yan et al., 2006). Activation of *FT1* can also bypass the normal requirement for vernalization; a strongly active allele of this gene was first identified as *Spring Growth Habit 3*, a locus linked to reduced vernalization requirement (subsequently named *VRN3*; Takahashi and Yasuda, 1971; Yan et al., 2006). This active allele has increased *FT1* gene copy number (four copies), which might cause increased transcriptional activity (Nitcher et al., 2013). A recent study suggests that structural alterations also occur amongst the four gene copies in this allele, so the exact cause of increased *FT1* transcription remains unclear (Loscos et al., 2014).

The FT1 protein of wheat interacts with FD-like proteins, analogous to FT and FD in *Arabidopsis* (Li and Dubcovsky, 2008). This is likely to activate expression of *AP1*-like genes at the shoot apex, including *VRN1* but also two other *AP1*-like genes (Preston and Kellogg, 2008). FT1 also interacts with FDL2 in leaves (Li and Dubcovsky, 2008). This potentially allows long-day induction of *VRN1* in leaves. This pathway operates in cereal varieties where activation of *FT1* triggers inflorescence initiation without prior vernalization, such as those that lack *VRN2* or that carry active *FT1* (*VRN3*) alleles (Yan et al., 2006; Hemming et al., 2008; Shimada et al., 2009). Long-day induction of *VRN1* via FT1 is unlikely to play a major role in triggering inflorescence initiation in response to vernalization, however, since *VRN1* is expressed at high levels in leaves and at the shoot apex of vernalized plants irrespective of daylength (Sasani et al., 2009). It is possible that a FT-FD dependent pathway might activate expression *VRN1* after short-day induction, or activate expression of other *AP1*-like genes in the leaves of vernalized plants.

Gibberellins play a role in the long-day flowering response of grasses (King and Evans, 2003). A shift to long days can trigger a rapid increase of gibberellin biosynthesis in leaves and application of gibberellins can accelerate inflorescence development in short days, mimicking the effect of longer daylengths (Evans et al., 1990; King et al., 2006). Leaf produced gibberellins might act as florigens in grasses, providing a mobile florigenic signal that is transported from the leaves to shoot apex to accelerate flowering, in addition to the FT protein (King and Evans, 2003). Applying gibberellins only accelerates inflorescence development when applied to vernalized plants or to accessions that carry active alleles of *VRN1*, placing the gibberellin response downstream of vernalization (MacMillan et al., 2005; Pearce et al., 2013; Boden et al., 2014). Paclobutrazol, an inhibitor of GA biosynthesis, slows the constitutive early flowering of *ELF3* loss-of-function mutants in short days without influencing *FT1* expression (Boden et al., 2014). This shows that gibberellins likely act parallel to, or downstream of, *FT1*.

### HIGH TEMPERATURES ACCELERATE INFLORESCENCE DEVELOPMENT IN LONG DAYS

Elevated growth temperatures can accelerate flowering of cereals. This occurs in long days, where elevated temperatures accelerate inflorescence development (Rawson and Richards, 1993). In short days high temperatures have the opposite effect, slowing inflorescence development (Rawson and Richards, 1993; Hemming et al., 2012). The acceleration of inflorescence development by high temperatures when days are long makes sense in a seasonal context, since this would further accelerate flowering toward the end of spring and allow grain production to occur more rapidly in warmer conditions that might accompany increased risk of heat stress and water limitation. Overall the physiology of the high-temperature flowering response in cereals is markedly different to that of *Arabidopsis*; accelerating inflorescence development post-initiation in long days, versus promoting floral initiation in short days.

Gene expression studies suggest that neither *FT1* nor other *FT*-like genes are high-temperature responsive in cereals (Hemming et al., 2012). Furthermore, activation of *FT1* by miss-expression of *PPD1* or by mutations in components of the circadian clock do not allow high-temperatures to accelerate inflorescence development in short-days (Hemming et al., 2012). This suggests that *FT1* is not the long-day activated factor that allows high-temperature acceleration of inflorescence development. Instead, it seems that an as yet unidentified long-day activated pathway allows high-temperatures to accelerate inflorescence development.

Transcriptome analyses have been used to identify high-temperature responsive developmental regulators in barley (Hemming et al., 2012). A series of genes thought to act downstream of *VRN1* in the vernalization response show altered expression at elevated temperatures, including the MADS box gene *ODDSOC2* (Hemming et al., 2012). This gene has no direct equivalent in *Arabidopsis* but might be related to the *FLC* gene family (Ruelens et al., 2013). *ODDSOC2* is down-regulated in vernalized barley plants and represses flowering when expressed constitutively, likely through down-regulation of *FLOWERING PROMOTER FACTOR1*-like genes (*FPF1*-like; Greenup et al., 2010). *ODDSOC2* is expressed at elevated levels when vernalized plants are grown at high temperatures (constant 25°C) in short-days, where inflorescence development is retarded (Hemming et al., 2012). This highlights a potentially interesting parallel with the role of *FLC*-like MADS box genes (*FLM*) in regulating temperature responses in *Arabidopsis* and other plants (Lee et al., 2013; Posé et al., 2013). *FPF1*-like genes, a potential downstream target of *ODDSOC2*, also show temperature-responsive expression in barley (Hemming et al., 2012). Further research is required to confirm a role for *ODDSOC2* and *FPF1*-like genes in regulating temperature-induced flowering responses of cereals.

### MAPPING MOLECULAR NETWORKS CONTROLLING SEASONAL FLOWERING: CONSIDERATIONS FOR CEREALS VERSUS *Arabidopsis*

**FIGURE 2:**
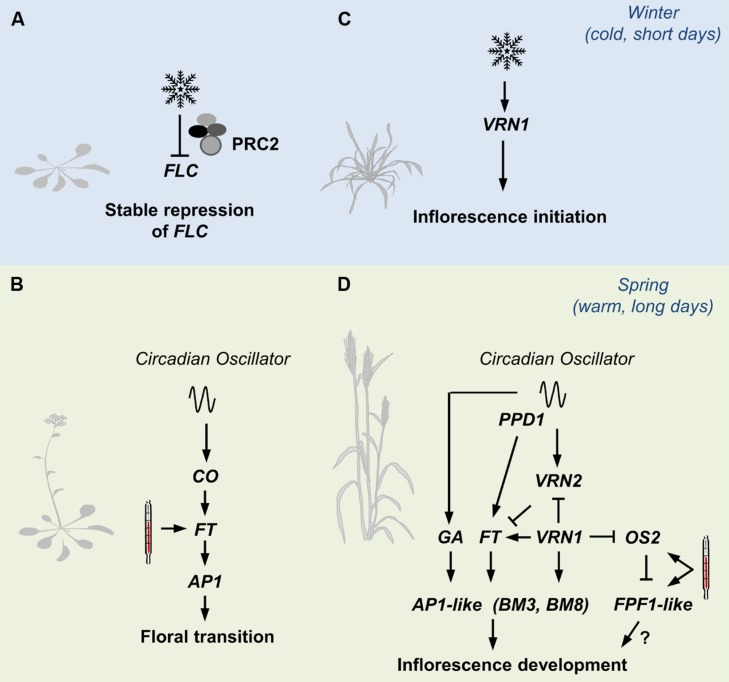
**An overview of pathways controlling seasonal flowering responses in grasses contrasted with those in *Arabidopsis*. (A)** The prolonged cold of winter (snowflake) triggers lasting repression of *FLOWERING LOCUS C* (*FLC*) in *Arabidopsis*, via the Polycomb Repressor Complex 2 (PRC2). **(B)** The long days of spring activate expression of *FLOWERING LOCUS T*, a process mediated by the circadian oscillator via *CONSTANS* (*CO*). *FT* activates expression of genes such as a *APETALA1* that trigger floral development. High-temperatures (thermometer) can also activate expression of *FT* to accelerate flowering. **(C)** Winter cold activates expression of *VERNALIZATION1* (*VRN1)* in cereals and related grasses. *VRN1* promotes inflorescence initiation at the shoot. **(D)** *VRN1* remains active after winter and down-regulates *VRN2*, which would otherwise repress the long-day flowering response in leaves. As daylength increases after winter, expression of *FT-like 1* is activated by the circadian oscillator, via *PHOTOPERIOD1*. Long days also activate gibberellin (GA) biosynthesis. The long-day flowering response activates expression of genes at the shoot apex that promote the development of floral organs. These include other *AP1*-like genes (*BM3* and *BM8* in barley for example). High temperatures accelerate the long-day response, possibly via *FLOWERING PROMOTER1-like* genes *(FPF1)*.

As outlined above, multiple pathways controlling developmental responses to seasonal cues are integrated to determine the timing of a single developmental transition in *Arabidopsis*. These pathways can be mapped together as a single network. The situation is more complex in grasses, where seasonal cues have varying effects at different developmental stages and/or different times of year. We suggest that molecular pathways controlling seasonal developmental responses in grasses should be considered in a developmental stage and season specific manner (**Figure 2**). The concept of sub-dividing networks controlling developmental responses to specific phases/seasons is also important for the development of gene based models to predict flowering behavior of cereals and related grasses (Brown et al., 2013).

## VARIATION IN FLOWERING BEHAVIOR ADAPTS CEREALS TO DIFFERENT GEOGRAPHICAL REGIONS

Diversity in the *VRN1* gene is a major driver of variation in vernalization requirement in temperate cereal crops. Different mutations in the promoter, insertions or deletions in the first intron, amino acid substitutions and copy number variation all occur at the *VRN1* locus (**Table 1**). Similar, though not identical, variation occurs in non-domesticated wheats, so variation in vernalization requirement mediated by *VRN1* occurs independently of crop domestication (Golovnina et al., 2010).

**Table 1:** Variation in the *VRN1* gene of cultivated cereals.

Variation	Genome	Effect	Reference
Promoter mutations, insertion deletions	A^m^	Elevated expression, Reduced vernalization req.	Yan et al. (2003)
Large deletions, first intron	A, B, D, H	Elevated expression, Reduced vernalization req.	Fu et al. (2005), Oliver et al. (2013)
Insertion, first intron	H	Elevated expression, Reduced vernalization req.	Cockram et al. (2007), Stockinger et al. (2007), Oliver et al. (2013)
Promoter SNP (and intron deletion)	D	Reduced expression, Increased vernalization req.	Zhang et al. (2012)
CNV (3 x), coding region SNP	A	Reduced expression, Increased vernalization req.	Chen et al. (2009), Díaz et al. (2012)
CNV (2 x) with promoter insertion	A	Elevated expression, Reduced vernalization req.	Yan et al. (2004b)

*A^m^ = Triticum monococcum.*

*H = Hordeum vulgare.*

*A, B, D = subgenomes of hexaploid bread wheat (Triticum aestivum).*

*CNV = copy number variation.*

The extent of diversity in *VRN1* suggests that variation in this gene might be useful to adapt accessions to different environments and there is strong historical evidence that this is the case. The history of the Australian wheat industry provides an excellent example. The first wheats grown in Australia were English wheats that required both vernalization and long days to flower rapidly. These wheats were ill-suited to warm Australian growing environments, taking too long to flower in the field, and suffered from end of season heat stress and water limitation, and also strong disease pressure (see Evans, 1980; Eagles et al., 2009). In the late 19th century William Farrer had the foresight to realize that selective breeding could be used to improve the adaptation of Australian wheats. He imported early flowering wheats from India, which he then crossed with European wheats to select better adapted strains. His first cultivar “Federation” was released in 1901 and proved enormously successful. Indeed, the germplasm he developed was the basis for adaptation of Australian wheat cultivars until the 1960s (see Eagles et al., 2009). Molecular characterization of the Australian wheat pedigree, using DNA extracted from seeds held in stock centers, shows that the reduced vernalization requirement of Federation was caused by an allele of *VRN1* that has a mutation in the promoter (Eagles et al., 2009).

Variation in *VRN1* can also increase the duration of vernalization required to trigger rapid flowering. A multi-copy allele of *VRN1*, with a substitution of a conserved amino acid residue in at least one copy, occurs in winter wheats grown in regions with cold winters (Chen et al., 2009; Díaz et al., 2012; Cane et al., 2013). This allele is associated with low-transcriptional activity and a slow transcriptional response to vernalization, and is linked to increased requirement for vernalization (Díaz et al., 2012). An association screen suggests that frost tolerant wheats have this allele of *VRN1* together with an expanded number of *C-REPEAT BINDING FACTOR* genes at the *FROST TOLERANCE 2* locus (Zhu et al., 2014). It seems that variation in *VRN1* allows the vegetative growth phase to be lengthened in cultivars grown in regions with extreme winters and this has been co-selected with genes that enhance frost tolerance.

There is also extensive variation in daylength sensitivity amongst cereals (**Table 2**). In wheat, a constitutive long-day flowering response is mediated mainly by active alleles of *PPD1* that allow crops to be grown at times of year or at latitudes where short daylengths would otherwise limit cultivation. For example, breeding programs that are located at low-latitudes and focus on rapid cycling varieties (e.g., International Maize and Wheat Improvement Centre, CIMMYT, Mexico) are likely to select cultivars with photoperiod insensitivity, which allows rapid flowering without long days. Conversely, a mutated version of the barley *PPD1* gene occurs in many European barleys (Turner et al., 2005; Jones et al., 2011). These barleys have a reduced response to long-days, which lengthens the growing season and allows more biomass to accumulate, increasing grain yield (see Cockram et al., 2007). Another example of adaptation through altered photoperiod sensitivity is the use of a loss-of-function mutation in the *ELF3* gene (MAT.a8) to breed barley cultivars that flower rapidly irrespective of daylength, which are well suited to short summer growing seasons at high latitudes (Lundqvist, 2009; Faure et al., 2012; Zakhrabekova et al., 2012).

**Table 2:** Variation in the *PPD1* gene of cultivated cereals.

Variation	Genome	Effect	Reference
Promoter deletion	A, D	Reduced long day requirement	Beales et al. (2007), Wilhelm et al. (2009), Shaw et al. (2013)
Promoter insertion	A	Reduced long day requirement	Nishida et al. (2013)
CNV (2–4.5 x)	B	Reduced long day requirement	Díaz et al. (2012)
CCT domain mutation	H	Reduced long-day response	Turner et al. (2005)
Promoter deletion	A	Reduced long-day response	Shaw et al. (2013)
Deletion, transcribed sequence	A	Reduced long-day response	Beales et al. (2007), Shaw et al. (2013)
Deletion, transcribed sequence	D	Reduced long-day response	Beales et al. (2007), Shaw et al. (2013)
Intron insertion	D	Reduced long-day response	Beales et al. (2007), Shaw et al. (2013)

*H = Hordeum vulgare.*

*A, B, D = subgenomes of hexaploid bread wheat (Triticum aestivum).*

*CNV = copy number variation.*

Variation that influences development irrespective of environmental cues, often referred to as the *EARLINESS PER SE* (*EPS*), has also been utilized in crop breeding. The *EPS2* gene (*HvCENTRORADIALIS, HvCEN*) of barley encodes a PEBP that is distantly related to the *FT* gene family (Comadran et al., 2012). An amino acid substitution in the *HvCEN* gene (Ala135 to Pro) delays flowering, and has been used to lengthen the inflorescence development phase of European spring barleys. Conversely, the wildtype allele predominates in autumn sown vernalization responsive barleys (Comadran et al., 2012). Thus, *HvCEN1* can be considered as a modifier of seasonal flowering responses.

**FIGURE 3:**
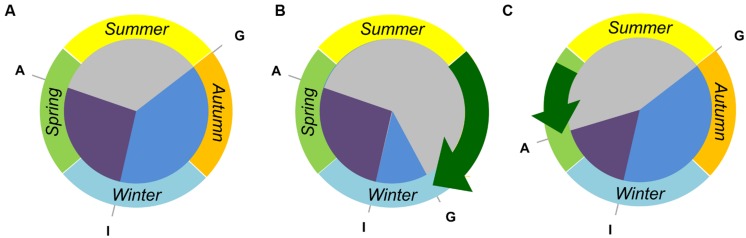
**Tailoring the crop life cycle to fit different environments by modifying the duration of discrete developmental phases. (A)** A vernalization responsive and daylength sensitive cereal cultivar can be sown and germinate “G” in early autumn but will remain vegetative until late winter, when inflorescence initiation occurs “I”. Anthesis “A” will occur during spring, at a date determined by the photoperiod sensitivity. **(B)** A near-isogenic line derived from the hypothetical wheat has reduced vernalization requirement. This line can be sown at a later date and will have a shortened vegetative growth phase (blue sector). Such a line might be useful in regions where there is a late onset of autumn rain, requiring a delayed sowing of crops. **(C)** A second near-isogenic line has reduced photoperiod sensitivity. This line can be sown in autumn, and still requires vernalization to flower, but will progress through inflorescence development (purple sector) more rapidly in spring. Such a line would be useful where there is a rapid onset of hot/dry conditions, allowing plants to avoid heat and drought stress during the grain filling stage.

The examples above highlight how different developmental regulators have been used to modify the crop life cycle to suit the growing environments encountered at particular location and sowing dates. These examples also show that such variation influences the duration of specific developmental phases (**Figure 3**). Altering the duration of different developmental phases can have profound effects on plant physiology and plant architecture, and strongly influences major components of yield such as grain number per spike and grain size (e.g., Stelmakh, 1993; Eagles et al., 2014). There is now considerable interest in understanding how different combinations of genetic variation in genes controlling flowering behavior can be used to breed cereal varieties that are suited to specific climates but that also have high yield potential (Reynolds et al., 2009).

## SEASONAL FLOWERING RESPONSES OF TEMPERATE GRASSES

Temperate grasses such as poa (*Poa* sp.), ryegrass (*Lolium* sp.), fescue (*Festuca* sp.), and timothy grass (*Phleum pretense*) flower in response to vernalization and long days, though there is naturally occurring variation in vernalization and daylength requirements both between species of specific genera and within populations of individual species (Evans, 1964). The molecular biology of seasonal flowering-responses has been studied in some temperate grasses. Orthologs of *VRN1* appear to function in the vernalization responses of perennial ryegrass (*Lolium perenne*), timothy grass (*Phleum pratense*), and fescue (*Festuca pratensis*; Petersen et al., 2004; Andersen et al., 2006; Seppänen et al., 2010; Ergon et al., 2013). The *VRN1* ortholog of *Brachypodium* is also likely to play a key role in maintaining a memory of winter cold (Ream et al., 2014). Similarly, *FT1*-like genes are likely to play a central role in promoting flowering in response to long days in perennial ryegrass and *Brachypodium* (King et al., 2006; Lv et al., 2014; Ream et al., 2014). Genetic variation in vernalization requirement or daylength sensitivity has also been linked to *VRN1* and *FT1*-like genes in perennial ryegrass (Jensen et al., 2005; Asp et al., 2011; Skøt et al., 2011; Shinozuka et al., 2013). Thus, knowledge of the molecular pathways controlling seasonal flowering responses in temperate cereals is relevant to Pooid grasses and can provide insights into how variation in genes controlling vernalization and daylength requirements can adapt temperate grasses to different climates or geographical regions.

### EVOLUTION OF SEASONAL FLOWERING-RESPONSES IN THE GRASSES

**FIGURE 4:**
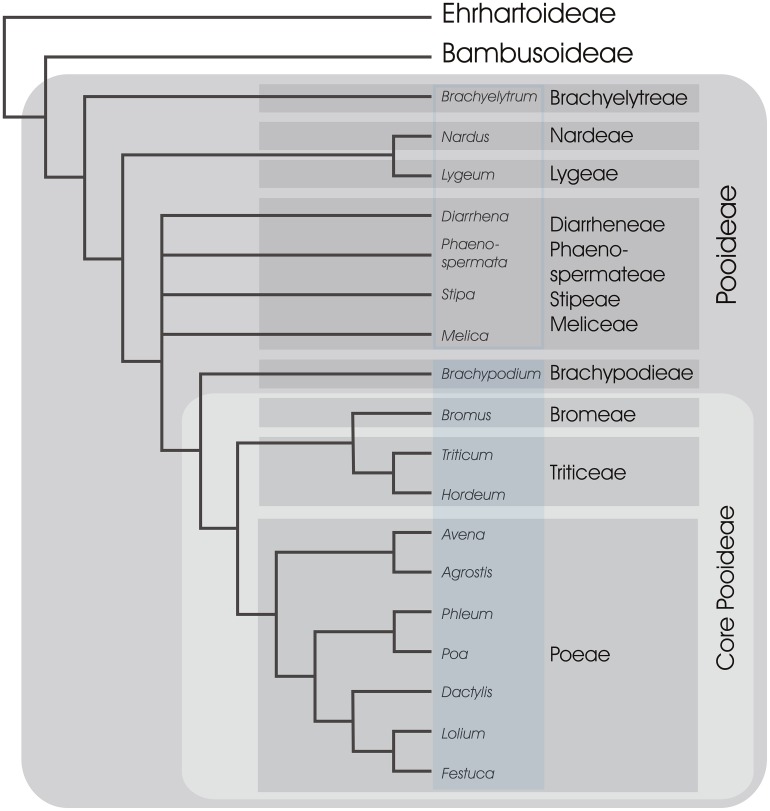
**A simplified phylogeny of subfamily Pooideae.** Relationships between major tribes of Pooid grasses, represented by selected genera, are shown based on Davis and Soreng (2007) and Schneider et al. (2009). Tribes are in shaded gray boxes and 11 out of 14 tribes are included following classification of Soreng et al. (2000). All genera represented in Brachypodieae, Bromeae, Triticeae, and Poeae are known to have at least some species flowering in response to vernalization and long days (shaded blue rectangle; Fejer, 1966; Sampson and Burrows, 1972; Richardson et al., 1986; Heide, 1994; Schwartz et al., 2010), whereas the remaining genera in Pooideae have unknown flowering requirements (open blue rectangle). The two closest non-Pooideae subfamilies (Ehrhartoideae and Bambusoideae) are also shown (BEP clade).

Most of the ∼10,000 species of the grass family are members of one of two monophyletic clades (groups of species including a single common ancestor and all its descendents). The first clade includes tropical grass subfamilies and is known as the PACMAD (Panicoideae, Arundinoideae, Chloridoideae, Micrairoideae, Aristidoideae, Danthonioideae), of which the Panicoideae subfamily includes crops such as maize (*Zea mays*), sorghum (*Sorghum bicolor*), and sugar cane (*Saccharum officinarum*). The second clade includes the Pooideae subfamily, together with the subfamilies Ehrhartoideae (including rice, *Oryza sativa*) and Bambusoideae (the bamboos) and is known as the BEP clade, with Bambusoideae widely accepted as the sister group to the Pooideae (**Figure 4**; Grass Phylogeny Working Group [GPWG] II, 2012; Wu and Ge, 2012; Zhao et al., 2013). Of the grass subfamilies, Pooideae is one of the most species rich and dominates the grass flora in temperate regions with as much as 90% of the grasses in northern temperate regions belonging to this subfamily (Hartley, 1973).

Current knowledge of the molecular pathways that control flowering of temperate grasses comes from a restricted selection of cereals and pasture grasses, all members of a group informally termed the “core Pooideae” (Davis and Soreng, 1993), or from *Brachypodium*, the sister group to the core Pooideae (**Figure 4**). The vernalization response and long-day flowering are conserved features of the core Pooideae and *Brachypodium* (**Figure 4**). In contrast, rice does not require vernalization and flowers in response to shorter daylengths, similar to many grasses of the Panicoideae. Thus, it has been hypothesized that the vernalization and long-day flowering responses of grasses evolved in an ancestor of the Pooideae.

### THE EVOLUTION OF THE VERNALIZATION RESPONSE

As outlined above, orthologs of the cereal *VRN1* gene function in the vernalization responses of temperate grasses such as ryegrass, fescue, and timothy grass (all core Pooids), and the *VRN1* gene of *Brachypodium* seems to have the same function. This suggests that the central role for *VRN1* in the vernalization response of temperate grasses evolved early during the radiation of the Pooideae. Vernalization-induced flowering likely co-evolved with increased freezing tolerance (see Sandve et al., 2011).

There are two regulatory features of *VRN1* that are likely to have been pivotal to the evolution of the vernalization response in temperate grasses. The first is that activity of *VRN1* is maintained at a low basal level before winter to delay inflorescence initiation. The second is low-temperature induction. Low basal activity of *VRN1* appears to be mediated by the large (∼10 kb) first intron, which has expanded relative to the equivalent region in the rice ortholog of *VRN1* (*OsMADS14*, ∼5 kb first intron; Fu et al., 2005; von Zitzewitz et al., 2005; Szûcs et al., 2007; Hemming et al., 2009; Oliver et al., 2013). Low-temperature induction of *VRN1* seems to be controlled by the promoter, possibly by pathways that activate transcription of other cold-induced genes (Alonso-Peral et al., 2011; Oliver et al., 2013). A deeper understanding how the first intron limits *VRN1* transcription, or of the mechanisms mediating low-temperature induction of *VRN1*, could potentially contribute to a better understanding of how the vernalization response evolved in grasses.

### THE EVOLUTION OF THE LONG-DAY FLOWERING RESPONSE

One explanation for the evolution of different daylength flowering responses is that the activity of the daylength response can be inverted. For example, the short-day flowering response of rice might have arisen through inversion of activity of the long-day response pathway first identified in *Arabidopsis*. This has been suggested to occur through modification of CO activity (see Simpson, 2003). A similar inversion in the daylength response could also be suggested to account for the evolution of long-day grasses from tropical grass ancestors that flowered in short days. We suggest, however, that complete inversions of daylength responses are unlikely to occur because daylength responses influence other aspects of plant physiology aside from flowering time. For example, daylength regulates the rate of starch breakdown during the night so that starch is depleted shortly before dawn (Graf and Smith, 2012). *CO* strongly influences expression of genes that control diurnal starch metabolism (Ortiz-Marchena et al., 2014). Unlike flowering behavior, daylength regulation of starch metabolism is likely to be conserved between short and long day grasses, which will need similar daylength dependent regulation of starch metabolism. This potentially limits evolutionary flexibility to invert daylength-induced flowering responses by modifying CO activity.

An alternative model is that a common daylength response mechanism elicits different outputs to trigger flowering under either short or long days in different plants. According to this model a conserved daylength response mechanism operates in all plants. Then, there are both short and long day output pathways that influence flowering, but these have evolved to be stronger or weaker in different lineages (Greenup et al., 2009). For example, the temperate grasses might have evolved to flower in long days through the loss of ancestral genes that accelerated flowering under short days (Greenup et al., 2009).

There are similarities between the molecular pathways controlling daylength responses of rice and temperate cereals that suggest there has not been an inversion of the activity of pathways controlling daylength-induced flowering responses. For example, a *VRN2*-like gene, *GHD7*, is expressed in long days in rice, where it represses expression of *FT*-like genes, similar to *VRN2* in the temperate cereals (Xue et al., 2008). In rice plants that lack *GHD7* function there is expression of *FT*-like genes in long-days and this accelerates flowering (Xue et al., 2008). Similarly, the maize (*Z. mays*) ortholog of *GHD7* represses flowering in long-days and a similar function has been suggested for a Sorghum (*S. bicolor*) equivalent (Ducrocq et al., 2009; Hung et al., 2012). Thus, a role for *VRN2*-like genes in blocking flowering under long-days occurs across the grass lineage, though is not necessarily related to a vernalization requirement. A recent study of sorghum shows that CO activates expression of *FT-like* genes in long-days in lines that lack *GHD7*, consistent with the idea that CO activity has not been inverted to repress flowering in long-days in tropical grasses (Yang et al., 2014). The weak inhibition of inflorescence initiation of temperate grasses in short-days also suggests that Pooid grasses retain ancestral pathways that allow flowering under short-days, though these pathways are weaker than the long-day response that is activated in vernalized plants (see Sections “Short Days Allow Inflorescence Initiation” and “The Prolonged Cold of Winter Promotes Inflorescence Initiation”).

Novel daylength regulators might also have evolved to suppress flowering in short-days in the temperate grasses. A novel modifier of the daylength flowering response has been identified in *Brachypodium*, where a daylength regulated microRNA (miR5200) down-regulates *FT1* and *FT2* in short days (Wu et al., 2013). This microRNA is expressed in leaves in short days, where it triggers cleavage of *FT1* and *FT2* mRNA molecules (Wu et al., 2013). Whether similar microRNA molecules are active in other temperate grasses or in cereal crops will be an interesting topic for future research.

### ARE PERENNIALS THE MISSING LINK?

Perenniality occurs throughout the Pooideae (Watson and Dallwitz, 1992) and it seems that monocarpic annual grasses have evolved repeatedly from perennial ancestors. This raises the question of whether evolution of the seasonal flowering responses of the Pooideae should be considered from perennial perspective?

Molecular studies of perennial temperate grasses are less common than those focussed on annual species. The best characterized are perennial ryegrass and timothy grass, which have both been the focus of physiological and molecular studies (MacMillan et al., 2005; Seppänen et al., 2010; Jokela et al., 2013). Like other members of the core Pooideae, perennial ryegrass flowers in response to vernalization and long-days, and *VRN1* and *FT1* homologs play key roles in each of these responses, respectively (Petersen et al., 2004; King et al., 2006). The perennial growth habit arises from the retention of vegetative meristems at the base of the plant during flowering, allowing vegetative growth to continue after a period of summer dormancy. It is not clear whether these buds do not respond to vernalization or whether there is a loss of the memory of vernalization.

Timothy grass is also a member of the core Pooideae that flowers in response to vernalization then long days (Seppänen et al., 2010). Unlike annual grasses, only a percentage of tillers flower in spring, allowing vegetative growth to continue even when some tillers flower. The reason why some tillers flower while others remain vegetative is unclear, but this is an important question for understanding the perennial growth habit of this grass. Timothy grass produces a second flush of elongating tillers in summer. Unlike the tillers produced in spring, the inflorescence of these tillers does not develop fully. Expression of *VRN1* is activated by vernalization in the spring tillers but is not maintained in the summer tillers (Seppänen et al., 2010). This might be one reason that inflorescence development stalls in summer tillers. An inability to maintain *VRN1* expression might also contribute to the perennial growth habit of timothy grass.

Another interesting finding from timothy grass is that expression of a *VRN2*-like gene increases as daylength increases following vernalization (Seppänen et al., 2010). This contrasts with the expression behavior of *VRN2* in other core Pooideae, where *VRN2* is repressed following vernalization. A similar observation has been made in *Brachypodium*, where a *VRN2*-like gene is activated by long days in vernalized plants (Ream et al., 2014). Perhaps *VRN2-*like genes have a broader role in perennial grasses to repress flowering in the long days of summer and to restore a vegetative growth habit before autumn.

## FUTURE DIRECTIONS

The Pooideae are a worthwhile target for the study natural variation in seasonal flowering responses. There are large collections of different accessions from many Pooid species and this provides a rich source of genetic diversity, from wild species and domesticated crops. Rapidly developing genomic resources are allowing this diversity to be explored. Surveying diversity alone will not provide mechanistic insights into the molecular pathways controlling flowering or how variation in these pathways influences adaptation to different climates. The development and study of focussed genetic resources including mapping populations, near-isogenic lines, mutants, and transgenic plants will be a priority for future research, since these tools can resolve gene function and assess the impact of genetic variation.

Studying the molecular pathways controlling seasonal flowering responses in a broader range of Pooid species, particularly grasses outside the core Pooid group, will provide a clearer view of how these pathways evolved. Research in to basal Pooids, such as *Stipa* or *Melica* for example (**Figure 4**), might provide some indication of when the vernalization and long day flowering responses evolved during the radiation of the Pooideae. Additionally, a deeper understanding of the pathways controlling flowering in perennial Pooids can potentially offer further insights into the functions of key genes. Most importantly, ongoing research focussed directly on the Pooideae is essential if we are to develop a deeper understanding the reproductive biology of this important group of plants.

## AUTHOR CONTRIBUTIONS

Siri Fjellheim, Scott Boden, and Ben Trevaskis wrote the manuscript.

## Conflict of Interest Statement

The authors declare that the research was conducted in the absence of any commercial or financial relationships that could be construed as a potential conflict of interest.

## References

[B1] Abe M. Kobayashi Y. Yamamoto S. Daimon Y. Yamaguchi A. Ikeda Y. (2005). FD, a bZIP protein mediating signals from the floral pathway integrator FT at the shoot apex. *Science* 309 1052–1056 10.1126/science.1115983 16099979

[B2] Allard V. Veisz O. Kõszegi B. Rousset M. Le Gouis J. Martre P. (2012). The quantitative response of wheat vernalization to environmental variables indicates that vernalization is not a response to cold temperature. *J. Exp. Bot.* 63 847–857 10.1093/jxb/err316 21994169

[B3] Alonso-Peral M. M. Oliver S. N. Casao M. C. Greenup A. A. Trevaskis B. (2011). The promoter of the cereal VERNALIZATION1 gene is sufficient for transcriptional induction by prolonged cold. *PLoS ONE* 6:e29456 10.1371/journal.pone.0029456 PMC3248443 22242122

[B4] Amasino R. (2010). Seasonal and developmental timing of flowering. *Plant J.* 61 1001–1013 10.1111/j.1365-313X.2010.04148.x 20409274

[B5] Andersen J. R. Jensen L. B. Asp T. Lübberstedt T. (2006). Vernalization response in perennial ryegrass (*Lolium perenne* L.) involves orthologues of diploid wheat (*Triticum monococcum*) VRN1 and rice (*Oryza sativa*) Hd1. *Plant Mol. Biol.* 60 481–484 10.1007/s11103-005-4815-1 16525886

[B6] Asp T. Byrne S. Gundlach H. Bruggmann R. Mayer K. F. Andersen J. R. (2011). Comparative sequence analysis of VRN1 alleles of *Lolium perenne* with the co-linear regions in barley, wheat, and rice. *Mol. Genet. Genomics* 286 433–437 10.1007/s00438-011-0654-8 22081040

[B7] Balasubramanian S. Sureshkumar S. Lempe J. Weigel D. (2006). Potent induction of *Arabidopsis thaliana* flowering by elevated growth temperature. *PLoS Genet.* 2:e106 10.1371/journal.pgen.0020106 PMC1487179 16839183

[B8] Barnard C. (1964). “Form and structure,” in *Grasses and Grasslands* ed. Barnard C. (New York: MacMillan) 47–72

[B9] Bastow R. Mylne J. S. Lister C. Lippman Z. Martienssen R. A. Dean C. (2004). Vernalization requires epigenetic silencing of FLC by histone methylation. *Nature* 427 164–167 10.1038/nature02269 14712277

[B10] Beales J. Turner A. Griffiths S. Snape J. Laurie D. A. (2007). A pseudo-response regulator is misexpressed in the photoperiod insensitive Ppd-D1a mutant of wheat (*Triticum aestivum* L.). *Theor. Appl. Genet.* 115 721–733 10.1007/s00122-007-0603-4 17634915

[B11] Ben-Naim O. Eshed R. Parnis A. Teper-Bamnolker P. Shalit A. Coupland G. (2006). The CCAAT binding factor can mediate interactions between CONSTANS-like proteins and DNA. *Plant J.* 46 462–476 10.1111/j.1365-313X.2006.02706.x 16623906

[B12] Blázquez M. A. Ahn J. H. Weigel D. (2003). A thermosensory pathway controlling flowering time in *Arabidopsis thaliana*. *Nat. Genet.* 33 168–171 10.1038/ng1085 12548286

[B13] Boden S. A. Weiss D. Ross J. J. Davies N. W. Trevaskis B. Chandler P. M. (2014). EARLY FLOWERING3 regulates flowering in spring barley by mediating gibberellin production and *FLOWERING LOCUS T* expression. *Plant Cell* 26 1557–1569 10.1105/tpc.114.123794 24781117 PMC4036571

[B14] Brown H. E. Jamieson P. D. Brooking I. R. Moot D. J. Huth N. I. (2013). Integration of molecular and physiological models to explain time of anthesis in wheat. *Ann. Bot.* 112 1683–1703 10.1093/aob/mct224 24220102 PMC3838551

[B15] Campoli C. Pankin A. Drosse B. Casao C. Davis S. J. von Korff M. (2013). HvLUX1 is a candidate gene underlying the early maturity 10 locus in barley: phylogeny, diversity, and interactions with the circadian clock and photoperiodic pathways. *New Phytol.* 199 1045–1059 10.1111/nph.12346 23731278 PMC3902989

[B16] Campoli C. Shtaya M. Davis S. J. von Korff M. (2012). Expression conservation within the circadian clock of a monocot: natural variation at barley Ppd-H1 affects circadian expression of flowering time genes, but not clock orthologs. *BMC Plant Biol.* 12:97 10.1186/1471-2229-12-97 PMC3478166 22720803

[B17] Cane K. Eagles H. A. Laurie D. A. Trevaskis B. Vallance N. Eastwood R. F. (2013). Ppd-B1 and Ppd-D1 and their effects in southern Australian wheat. *Crop Pasture Sci.* 64 100–114 10.1071/CP13086

[B18] Casao M. C. Igartua E. Karsai I. Lasa J. M. Gracia M. P. Casas A. M. (2011). Expression analysis of vernalization and day-length response genes in barley (*Hordeum vulgare L*.) indicates that VRNH2 is a repressor of PPDH2 (HvFT3) under long days. *J. Exp. Bot.* 62 1939–1949 10.1093/jxb/erq382 21131547 PMC3060678

[B19] Chen A. Dubcovsky J. (2012). Wheat TILLING mutants show that the vernalization gene VRN1 down-regulates the flowering repressor VRN2 in leaves but is not essential for flowering. *PLoS Genet.* 8:e1003134 10.1371/journal.pgen.1003134 PMC3521655 23271982

[B20] Chen Y. Carver B. F. Wang S. Zhang F. Yan L. (2009). Genetic loci associated with stem elongation and winter dormancy release in wheat. *Theor. Appl. Genet.* 118 881–889 10.1007/s00122-008-0946-5 19130033

[B21] Chouard P. (1960). Vernalization and its relations to dormancy. *Annu. Rev. Plant Physiol.* 11 191–238 10.1146/annurev.pp.11.060160.001203

[B22] Cockram J. Chiapparino E. Taylor S. Stamati K. Donini P. Laurie D. (2007). Haplotype analysis of vernalization loci in European barley germplasm reveals novel VRN-H1 alleles and a predominant winter VRN-H1/VRN-H2 multi-locus haplotype. *Theor. Appl. Genet.* 115 993–1001 10.1007/s00122-007-0626-x 17713756

[B23] Comadran J. Kilian B. Russell J. Ramsay L. Stein N. Ganal M. (2012). Natural variation in a homolog of Antirrhinum CENTRORADIALIS contributed to spring growth habit and environmental adaptation in cultivated barley. *Nat. Genet.* 44 1388–1392 10.1038/ng.2447 23160098

[B24] Corbesier L. Vincent C. Jang S. H. Fornara F. Fan Q. Z. Searle I. (2007). FT protein movement contributes to long distance signaling in floral induction of *Arabidopsis*. *Science* 316 1030–1033 10.1126/science.1141752 17446353

[B25] Danyluk J. Kane N. A. Breton G. Limin A. E. Fowler D. B. Sarhan F. (2003). TaVRT-1, a putative transcription factor associated with vegetative to reproductive transition in cereals. *Plant Physiol.* 132 1849–1860 10.1104/pp.103.023523 12913142 PMC181271

[B26] Davis J. I. Soreng R. J. (1993). Phylogenetic structure in the grass family (Poaceae) as inferred from chloroplast DNA restriction site variation. *Am. J. Bot.* 80 1444–1454 10.2307/2445674

[B27] Davis J. I. Soreng R. J. (2007). A preliminary phylogenetic analysis of the grass subfamily Pooideae (Poaceae), with attention to structural features ofthe plastid and nuclear genomes, including an intron loss in GBSSI. *Aliso* 23 335–348 10.5642/aliso.20072301.27

[B28] Díaz A. Zikhali M. Turner A. S. Isaac P. Laurie D. A. (2012). Copy number variation affecting the Photoperiod-B1 and Vernalization-A1 genes is associated with altered flowering time in wheat (*Triticum aestivum*). *PLoS ONE* 7:e33234 10.1371/journal.pone.0033234 PMC3310869 22457747

[B29] Dubcovsky J. Chen C. Yan L. (2005). Molecular characterization of the allelic variation at the VRN-H2 vernalization locus in barley. *Mol. Breed.* 15 395–407 10.1007/s11032-005-0084-6

[B30] Dubcovsky J. Loukoianov A. Fu D. Valarik M. Sanchez A. Yan L. (2006). Effect of photoperiod on the regulation of wheat vernalization genes VRN1 and VRN2. *Plant Mol. Biol.* 60 469–480 10.1007/s11103-005-4814-2 16525885 PMC4739792

[B31] Ducrocq S. Giauffret C. Madur D. Combes V. Dumas F. Jouanne S. (2009). Fine mapping and haplotype structure analysis of a major flowering time quantitative trait locus on maize chromosome 10. *Genetics* 183 1555–1563 10.1534/genetics.109.106922 19822732 PMC2787439

[B32] Eagles H. A. Cane K. Vallance N. (2009). The flow of alleles of important photoperiod and vernalisation genes through Australian wheat. *Crop Pasture Sci.* 60 646–657 10.1071/CP09014

[B33] Eagles H. A. Cane K. Trevaskis B. Vallance N. Eastwood R. F. Gororo N. N. (2014). Ppd1, Vrn1, ALMT1 and Rht genes and their effects on grain yield in lower rainfall environments in southern Australia. *Crop Pasture Sci.* 65 159–170 10.1071/CP13374

[B34] Ergon Å. Hamland H. Rognli O. A. (2013). Differential expression of VRN1 and other MADS-box genes in *Festuca pratensis* selections with different vernalization requirements. *Biol. Plant.* 57: 245–254 10.1007/s10535-012-0283-z

[B35] Evans L. T. (1964). “Reproduction,” in *Grasses and Grasslands* ed. Barnard C. (New York: MacMillan) 126–153

[B36] Evans L. T. (1980). Response to challenge: William Farrer and the making of wheats. *J. Aust. Inst. Agric. Sci.* 46 3–13

[B37] Evans L. T. (1987). Short day induction of inflorescence initiation in some winter wheat varieties. *Funct. Plant Biol.* 14 277–286 10.1071/PP9870277

[B38] Evans L. T. King R. W. Chu A. Mander L. N. Pharis R. P. (1990). Gibberellin structure and florigenic activity in *Lolium temulentum*, a long-day plant. *Planta* 182 97–106 10.1007/BF00239990 24197004

[B39] Faure S. Higgins J. Turner A. Laurie D. A. (2007). The *FLOWERING LOCUS T*-like gene family in barley (*Hordeum vulgare*). *Genetics* 176 599–609 10.1534/genetics.106.069500 17339225 PMC1893030

[B40] Faure S. Turner A. S. Gruszka D. Christodoulou V. Davis S. J. von Korff M. (2012). Mutation at the circadian clock gene EARLY MATURITY 8 adapts domesticated barley (*Hordeum vulgare*) to short growing seasons. *Proc. Natl. Acad. Sci. U.S.A.* 109 8328–8333 10.1073/pnas.1120496109 22566625 PMC3361427

[B41] Fejer S. O. (1966). Growth and reproduction of New Zealand Mediterranean and hybrid *Dactylis glomerata* after short day and temperature treatments. *Can. J. Plant Sci.* 46 233–241 10.4141/cjps66-040

[B42] Finnegan E. J. Dennis E. S. (2007). vernalization-induced trimethylation of histone H3 Lysine 27 at FLC is not maintained in mitotically quiescent cells. *Curr. Biol.* 17 1978–1983 10.1016/j.cub.2007.10.026 17980595

[B43] Fowler D. B. Breton G. Limin A. E. Mahfoozi S. Sarhan F. (2001). Photoperiod and temperature interactions regulate low-temperature induced gene expression in barley. *Plant Physiol.* 127 1676–1681 10.1104/pp.010483 11743112 PMC133572

[B44] Fowler D. B. Chauvin L. P. Limin A. E. Sarhan F. (1996). The regulatory role of vernalization in the expression of low-temperature-induced genes in wheat and rye. *Theor. Appl. Genet.* 93 554–559 10.1007/BF00417947 24162347

[B45] Fu D. Szucs P. Yan L. Helguera M. Skinner J. S. von Zitzewitz J. (2005). Large deletions within the first intron in VRN-1 are associated with spring growth habit in barley and wheat. *Mol. Genet. Genomics* 273 54–65 10.1007/s00438-004-1095-4 15690172

[B46] Gassner G. (1918). Beitraege zur physiologischen charakteristik sommer und winterannueller gewaechse in besondere der getreidepflanzen. *Z. Bot.* 10 27–476 10.1007/BF00709593

[B47] Gawroñski P. Ariyadasa R. Himmelbach A. Poursarebani N. Kilian B. Stein N. (2014). A distorted circadian clock causes early flowering and temperature-dependent variation in spike development in the Eps-3Am mutant of einkorn wheat. *Genetics* 196 1253–1261 10.1534/genetics.113.158444 24443443 PMC3982698

[B48] Golovnina K. A. Kondratenko E. Y. Blinov A. G. Goncharov N. P. (2010). Molecular characterization of vernalization loci VRN1 in wild and cultivated wheats. *BMC Plant Biol.* 10:168 10.1186/1471-2229-10-168 PMC3095301 20699006

[B49] Gott M. B. Gregory F. G. Purvis O. N. (1955). Studies in vernalization of cereals VIII. photoperiodic control of stage in flowering between initiation and ear formation in vernalised and unvernalised petkus winter rye. *Ann. Bot.* 21 87–126

[B50] Graf A. Smith A. M. (2012). Starch and the clock: the dark side of plant productivity. *Trends Plant Sci.* 16 169–175 10.1016/j.tplants.2010.12.003 21216654

[B51] Grass Phylogeny Working Group [GPWG] II. (2012). New grass phylogeny resolves deep evolutionary relationships and discovers C4 origins. *New Phytol.* 193 304–312 10.1111/j.1469-8137.2011.03972.x 22115274

[B52] Greenup A. Peacock W. J. Dennis E. S. Trevaskis B. (2009). The molecular biology of seasonal flowering-responses in *Arabidopsis* and the cereals. *Ann. Bot.* 103 1165–1172 10.1093/aob/mcp063 19304997 PMC2685306

[B53] Greenup A. G. Sasani S. Oliver S. N. Talbot M. J. Dennis E. S. Hemming M. N. (2010). ODDSOC2 is a MADS-box floral repressor that is down-regulated by vernalization in temperate cereals. *Plant Physiol.* 153 1062–1073 10.1104/pp.109.152488 20431086 PMC2899939

[B54] Greenup A. G. Sasani S. Oliver S. N. Walford S. A. Millar A. A. Trevaskis B. (2011). Transcriptome analysis of the vernalization response in barley (*Hordeum vulgare*) seedlings. *PLoS ONE* 6:e17900 10.1371/journal.pone.0017900 PMC3052371 21408015

[B55] Hartley W. (1973). Studies on origin, evolution, and distribution of Gramineae. V. Subfamily Festucoideae. *Aust. J. Bot.* 21 201–234 10.1071/BT9730201

[B56] Heide O. M. (1994). Control of flowering and reproduction in temperate grasses. *New Phytol.* 128 347–362 10.1111/j.1469-8137.1994.tb04019.x 33874362

[B57] Helliwell C. A. Wood C. C. Robertson M. Peacock W. J. Dennis E. S. (2006). The Arabidopsis FLC protein interacts directly in vivo with SOC1 and FT chromatin and is part of a high-molecular weight protein complex. *Plant J.* 46 183–192 10.1111/j.1365-313X.2006.02686.x 16623882

[B58] Hemming M. N. Fieg S. Peacock W. J. Dennis E. S. Trevaskis B. (2009). Regions associated with repression of the barley (*Hordeum vulgare*) VERNALIZATION1 gene are not required for cold induction. *Mol. Genet. Genomics* 282 107–117 10.1007/s00438-009-0449-3 19404679

[B59] Hemming M. N. Peacock W. J. Dennis E. S. Trevaskis B. (2008). Low-temperature and daylength cues are integrated to regulate *FLOWERING LOCUS T* in barley. *Plant Physiol.* 147 355–366 10.1104/pp.108.116418 18359843 PMC2330320

[B60] Hemming M. N. Walford S. A. Fieg S. Dennis E. S. Trevaskis B. (2012). Identification of high-temperature-responsive genes in cereals. *Plant Physiol.* 158 1439–1450 10.1104/pp.111.192013 22279145 PMC3291267

[B61] Hung H. Y. Shannon L. M. Tian F. Bradbury P. J. Chen C. Flint-Garcia S. A. (2012). ZmCCT and the genetic basis of day-length adaptation underlying the postdomestication spread of maize. *Proc. Natl. Acad. Sci. U.S.A.* 109 E1913–E1921 10.1073/pnas.1203189109 22711828 PMC3396540

[B62] Jensen L. B. Andersen J. R. Frei U. Xing Y. Taylor C. Holm P. B. (2005). QTL mapping of vernalization response in perennial ryegrass (*Lolium perenne L*.) reveals co-location with an orthologue of wheat VRN1. *Theor. Appl. Genet.* 110 527–536 10.1007/s00122-004-1865-8 15619078

[B63] Jokela V. Virkajärvi P. Tanskanen J. Seppänen M. M. (2013). Vernalization, gibberellic acid and photo period are important signals of yield formation in timothy (*Phleum pratense*). *Physiol. Plant.* 10.1111/ppl.12141 [Epub ahead of print] 24329752

[B64] Jones H. Civáň P. Cockram J. Leigh F. J. Smith L. M. Jones M. K. (2011). Evolutionary history of barley cultivation in Europe revealed by genetic analysis of extant landraces. *BMC Evol. Biol.* 11:320 10.1186/1471-2148-11-320 PMC3248229 22047039

[B65] Kardailsky I. Shukla V. K. Ahn J. H. Dagenais N. Christensen S. K. Nguyen J. T. (1999). Activation tagging of the floral inducer FT. *Science* 286 1962–1965 10.1126/science.286.5446.1962 10583961

[B66] Karsai I. Szûcs P. Meszaros K. Filichkina T. Hayes P. M. Skinner J. S. (2005). The Vrn-H2 locus is a major determinant of flowering time in a facultative x winter growth habit barley (*Hordeum vulgare* L.) mapping population. *Theor. Appl. Genet.* 110 1458–1466 10.1007/s00122-005-1979-7 15834697

[B67] Kikuchi R. Kawahigashi H. Ando T. Tonooka T. Handa H. (2009). Molecular and functional characterization of PEBP genes in barley reveal the diversification of their roles in flowering. *Plant Physiol.* 149 1341–1353 10.1104/pp.108.132134 19168644 PMC2649388

[B68] King R. W. Evans L. T. (2003). Gibberellins and flowering of grasses and cereals: prizing open the lid of the “florigen” black box. *Annu. Rev. Plant Biol.* 54 307–328 10.1146/annurev.arplant.54.031902.135029 14502993

[B69] King R. W. Moritz T. Evans L. T. Martin J. Andersen C. H. Blundell C. (2006). Regulation of flowering in the long-day grass Lolium temulentum by gibberellins and the *FLOWERING LOCUS T* gene. *Plant Physiol.* 141 498–507 10.1104/pp.106.076760 16581877 PMC1475477

[B70] Kobayashi Y. Kaya H. Goto K. Iwabuchi M. Araki T. (1999). A pair of related genes with antagonistic roles in mediating flowering signals. *Science* 286 1960–1962 10.1126/science.286.5446.1960 10583960

[B71] Kuchel H. Williams K. J. Langridge P. Eagles H. A. Jefferies S. P. (2007). Genetic dissection of grain yield in bread wheat. I. QTL analysis. *Theor. Appl. Genet.* 115 1015–1027 10.1007/s00122-007-0628-8 17712541

[B72] Kumar S. V. Lucyshyn D. Jaeger K. E. Alós E. Alvey E. Harberd N. P. (2012). Transcription factor PIF4 controls thermosensory activation of flowering. *Nature* 484 242–245 10.1038/nature10928 22437497 PMC4972390

[B73] Lee H. Suh S. S. Park E. Cho E. Ahn J. H. Kim S. G. (2000). The AGAMOUS-LIKE 20 MADS domain protein integrates floral inductive pathways in *Arabidopsis*. *Genes Dev.* 15 2366–2376 10.1101/gad.813600 10995392 PMC316936

[B74] Lee J. H. Ryu H. S. Chung K. S. Posé D. Kim S. Schmid M. (2013). Regulation of temperature-responsive flowering by MADS-box transcription factor repressors. *Science* 342 628–632 10.1126/science.1241097 24030492

[B75] Li C. Distelfeld A. Comis A. Dubcovsky J. (2011). Wheat flowering repressor VRN2 and promoter CO_2_ compete for interactions with NUCLEAR FACTOR-Y complexes. *Plant J.* 67 763–773 10.1111/j.1365-313X.2011.04630.x 21554456 PMC4765905

[B76] Li C. Dubcovsky J. (2008). Wheat FT protein regulates VRN1 transcription through interactions with FDL2. *Plant J.* 55 543–554 10.1111/j.1365-313X.2008.03526.x 18433437 PMC4739743

[B77] Limin A. E. Fowler D. B. (2006). Low-temperature tolerance and genetic potential in wheat (*Triticum aestivum* L.): response to photoperiod, vernalization, and plant development. *Planta* 224 360–366 10.1007/s00425-006-0219-y 16440213

[B78] Loscos J. Igartua E. Contreras-Moreira B. Gracia M. P. Casas A. M. (2014). HvFT1 polymorphism and effect-survey of barley germplasm and expression analysis. *Front. Plant Sci.* 5:251 10.3389/fpls.2014.00251 PMC4047512 24936204

[B79] Lundqvist U. (2009). “Eighty years of Scandinavian barley mutation genetics and breeding,” in *Induced Plant Mutations in the Genomics Era* ed. Shu Q. Y. (Rome: Food and Agriculture Organization of the United Nations) 39–43

[B80] Lv B. Nitcher R. Han X. Wang S. Ni F. Li K. (2014). Characterization of *FLOWERING LOCUS T1* (FT1) gene in *Brachypodium* and wheat. *PLoS ONE* 9:e94171 10.1371/journal.pone.0094171 PMC3981775 24718312

[B81] MacMillan C. P. Blundell C. A. King R. W. (2005). Flowering of the grass *Lolium perenne*: effects of vernalization and long days on gibberellin biosynthesis and signaling. *Plant Physiol.* 138 1794–1806 10.1104/pp.105.062190 15980191 PMC1176447

[B82] Michaels S. D. Amasino R. M. (1999). *FLOWERING LOCUS C* encodes a novel MADS domain protein that acts as a repressor of flowering. *Plant Cell* 11 949–956 10.2307/3870827 10330478 PMC144226

[B83] Michaels S. D. Himelblau E. Kim S. Y. Schomburg F. M. Amasino R. M. (2005). Integration of flowering signals in winter-annual *Arabidopsis*. *Plant Physiol.* 137 376–385 10.1104/pp.104.052811 PMC548846 15618421

[B84] Miralles D. J. Richards R. A. (2000). Response of leaf and tiller emergence and primordium initiation in wheat and barley to interchanged photoperiod. *Ann. Bot.* 85 655–663 10.1006/anbo.2000.1121

[B85] Mizuno N. Nitta M. Sato K. Nasuda S. (2012). A wheat homologue of PHYTOCLOCK 1 is a candidate gene conferring the early heading phenotype to einkorn wheat. *Genes Genet. Syst.* 87 357–367 10.1266/ggs.87.357 23558642

[B86] Murai K. Miyamae M. Kato H. Takumi S. Ogihara Y. (2003). WAP1, a wheat APETALA1 homolog, plays a central role in the phase transition from vegetative to reproductive growth. *Plant Cell Physiol.* 44 1255–1265 10.1093/pcp/pcg171 14701921

[B87] Nishida H. Yoshida T. Kawakami K. Fujita M. Long B. Akashi Y. (2013). Structural variation in the 5^′^ upstream region of photoperiod-insensitive alleles Ppd-A1a and Ppd-B1a identified in hexaploid wheat (*Triticum aestivum* L.), and their effect on heading time. *Mol. Breed.* 31 27–37 10.1007/s11032-012-9765-0

[B88] Nitcher R. Distelfeld A. Tan C. Yan L. Dubcovsky J. (2013). Increased copy number at the HvFT1 locus is associated with accelerated flowering time in barley. *Mol. Genet. Genomics* 288 261–275 10.1007/s00438-013-0746-8 23591592 PMC3664738

[B89] Oliver S. N. Deng W. Casao M. C. Trevaskis B. (2013). Low temperatures induce rapid changes in chromatin state and transcript levels of the cereal VERNALIZATION1 gene. *J. Exp. Bot.* 64 2413–2422 10.1093/jxb/ert095 23580755 PMC3654426

[B90] Oliver S. N. Finnegan E. J. Dennis E. S. Peacock W. J. Trevaskis B. (2009). Vernalization-induced flowering in cereals is associated with changes in histone methylation at the VERNALIZATION1 gene. *Proc. Natl. Acad. Sci. U.S.A.* 106 8386–8391 10.1073/pnas.0903566106 19416817 PMC2677093

[B91] Ortiz-Marchena M. I. Albi T. Lucas-Reina E. Said F. E. Romero-Campero F. J. Cano B. (2014). Photoperiodic control of carbon distribution during the floral transition in *Arabidopsis*. *Plant Cell* 26 565–584 10.1105/tpc.114.122721 24563199 PMC3967026

[B92] Pearce S. Vanzetti L. S. Dubcovsky J. (2013). Exogenous gibberellins induce wheat spike development under short days only in the presence of VERNALIZATION1. *Plant Physiol.* 163 1433–1445 10.1104/pp.113.225854 24085801 PMC3813662

[B93] Petersen K. Didion T. Andersen C. H. Nielsen K. K. (2004). MADS-box genes from perennial ryegrass differentially expressed during transition from vegetative to reproductive growth. *J. Plant Physiol.* 161 439–447 10.1078/0176-1617-01212 15128031

[B94] Posé D. Verhage L. Ott F. Yant L. Mathieu J. Angenent G. C. (2013). Temperature-dependent regulation of flowering by antagonistic FLM variants. *Nature* 503 414–417 10.1038/nature12633 24067612

[B95] Preston J. C. Kellogg E. A. (2008). Discrete developmental roles for temperate cereal grass VERNALIZATION1/FRUITFULL-Like genes in flowering competency and the transition to flowering. *Plant Physiol.* 146 265–276 10.1104/pp.107.109561 18024551 PMC2230560

[B96] Purvis O. N. (1934). An analysis of the influence of temperature during germination on the subsequent development of certain winter cereals and its relation to the effect of length of day. *Ann. Bot.* 48 919–955

[B97] Purvis O. N. Gregory F. G. (1937). Studies in vernalisation of cereals. *Ann. Bot.* 1 1–26

[B98] Rawson H. M. Richards R. A. (1993). Effects of high temperature and photoperiod on floral development in wheat isolines differing in vernalisation and photoperiod genes. *Field Crops Res.* 32 181–192 10.1016/0378-4290(93)90030-Q

[B99] Ream T. S. Woods D. P. Schwartz C. J. Sanabria C. P. Mahoy J. A. Walters E. M. (2014). Interaction of photoperiod and vernalization determines flowering time of *Brachypodium distachyon*. *Plant Physiol.* 164 694–709 10.1104/pp.113.232678 24357601 PMC3912099

[B100] Reynolds M. Foulkes M. J. Slafer G. A. Berry P. Parry M. A. Snape J. W. (2009). Raising yield potential in wheat. *J. Exp. Bot.* 60 1899–1918 10.1093/jxb/erp016 19363203

[B101] Richardson J. M. Morrow L. A. Gealy D. R. (1986). Floral induction of downy brome (*Bromus tectorum*) as influenced by temperature and photoperiod. *Weed Sci.* 34 698–703

[B102] Ruelens P. de Maagd R. A. Proost S. Theissen G. Geuten K. Kaufmann K. (2013). *FLOWERING LOCUS C* in monocots and the tandem origin of angiosperm-specific MADS-box genes. *Nat. Commun.* 4 1–8 10.1038/ncomms3280 23955420

[B103] Sampson D. R. Burrows V. D. (1972). Influence of photoperiod, short-day vernalization, and cold vernalization on days to heading in *Avena* species and cultivars. *Can. J. Plant Sci.* 52 471–482 10.4141/cjps72-077

[B104] Sandve S. R. Kosmala A. Rudi H. Fjellheim S. Rapacz M. Yamada T. (2011). Molecular mechanisms underlying frost tolerance in perennial grasses adapted to cold climates. *Plant Sci.* 180 69–77 10.1016/j.plantsci.2010.07.011 21421349

[B105] Sasani S. Hemming M. N. Oliver S. N. Greenup A. Tavakkol-Afshari R. Mahfoozi S. (2009). The influence of vernalization and daylength on expression of flowering-time genes in the shoot apex and leaves of barley (*Hordeum vulgare*). *J. Exp. Bot.* 60 2169–2178 10.1093/jxb/erp098 19357429 PMC2682508

[B106] Schneider J. Doring E. Hilu K. W. Roser M. (2009). Phylogenetic structure of the grass subfamily Pooideae based on comparison of plastid matK gene-3^′^ trnK exon and nuclear ITS sequences. *Taxon* 58 405–424

[B107] Schubert D. Primavesi L. Bishopp A. Roberts G. Doonan J. Jenuwein T. (2006). Silencing by plant Polycomb-group genes requires dispersed trimethylation of histone H3 at lysine 27. *EMBO J.* 25 4638–4649 10.1038/sj.emboj.7601311 16957776 PMC1590001

[B108] Schwartz C. J. Doyle M. R. Manzaneda A. J. Rey P. J. Mitchell-Olds T. Amasino R. M. (2010). Natural variation of flowering time and vernalization responsiveness in *Brachypodium distachyon*. *Bioenergy Res.* 3 38–46 10.1007/s12155-009-9069-9063

[B109] Seppänen M. M. Pakarinen K. Jokela V. Andersen J. R. Fiil A. Santanen A. (2010). Vernalization response of *Phleum pratense* and its relationships to stem lignification and floral transition. *Ann. Bot.* 106 697–707 10.1093/aob/mcq174 20798263 PMC2958789

[B110] Shaw L. M. Turner A. S. Herryl L. Griffiths S. Laurie D. A. (2013). Mutant alleles of Photoperiod-1 in wheat (*Triticum aestivum* L.) that confer a late flowering phenotype in long days. *PLoS ONE* 8:e79459 10.1371/journal.pone.0079459 PMC3828349 24244507

[B111] Shaw L. M. Turner A. S. Laurie D. A. (2012). The impact of photoperiod insensitive Ppd-1a mutations on the photoperiod pathway across the three genomes of hexaploid wheat (*Triticum aestivum*). *Plant J.* 71 71–84 10.1111/j.1365-313X.2012.04971.x 22372488

[B112] Sheldon C. C. Burn J. E. Perez P. P. Metzger J. Edwards J. A. Peacock W. J. (1999). The FLF MADS box gene: a repressor of flowering in *Arabidopsis* regulated by vernalization and methylation. *Plant Cell* 11 445–458 10.2307/3870872 10072403 PMC144185

[B113] Shimada S. Ogawa T. Kitagawa S. Suzuki T. Ikari C. Shitsukawa N. (2009). A genetic network of flowering-time genes in wheat leaves, in which an APETALA1/FRUITFULL-like gene, VRN1, is upstream of *FLOWERING LOCUS T*. *Plant J.* 58 668–681 10.1111/j.1365-313X.2009.03806.x 19175767 PMC2721963

[B114] Shinozuka H. Hand M. L. Cogan N. O. Spangenberg G. C. Forster J. W. (2013). Nucleotide diversity of vernalization and flowering-time-related genes in a germplasm collection of meadow fescue (*Festuca pratensis* Huds. syn. *Lolium pratense* (Huds.) Darbysh.). *Evol. Ecol.* 3 4415–4426 10.1002/ece3.828 PMC3856742 24340183

[B115] Simpson G. G. (2003). Evolution of flowering in response to day length: flipping the CONSTANS switch. *Bioessays* 25 829–832 10.1002/bies.10330 12938171

[B116] Skøt L. Sanderson R. Thomas A. Skøt K. Thorogood D. Latypova G. (2011). Allelic variation in the perennial ryegrass *FLOWERING LOCUS T* gene is associated with changes in flowering time across a range of populations. *Plant Physiol.* 155 1013–1022 10.1104/pp.110.169870 21115808 PMC3032449

[B117] Soreng R. J. Davidse G. Peterson P. M. Zuloaga F. O. Judziewicz E. J. Filgueiras T. S. (2000). *Classification of New World Grasses (Poaceae/Gramineae).* Available at: http://www.tropicos.org/projectwebportal.aspx?pagename=ClassificationNWG&projectid=10

[B118] Stelmakh A. F. (1993). Genetic effects of Vrn genes on heading date and agronomic traits in bread wheat. *Euphytica* 65 53–60 10.1007/BF00022199

[B119] Stockinger E. J. Skinner J. S. Gardner K. G. Francia E. Pecchioni N. (2007). Expression levels of barley Cbf genes at the Frost resistance-H2 locus are dependent upon alleles at Fr-H1 and Fr-H2. *Plant J.* 51 308–321 10.1111/j.1365-313X.2007.0141.x 17559507

[B120] Suarez-Lopez P. Wheatley K. Robson F. Onouchi H. Valverde F. Coupland G. (2001). CONSTANS mediates between the circadian clock and the control of flowering in *Arabidopsis*. *Nature* 410 1116–1120 10.1038/35074138 11323677

[B121] Szûcs P. Skinner J. S. Karsai I. Cuesta-Marcos A. Haggard K. G. Corey A. E. (2007). Validation of the VRN-H2/VRN-H1 epistatic model in barley reveals that intron length variation in VRN-H1 may account for a continuum of vernalization sensitivity. *Mol. Genet. Genomics* 277 249–261 10.1007/s00438-006-0195-8 17151889

[B122] Takahashi R. Yasuda S. (1971). “Genetics of earliness and growth habit in barley,” in *Barley Genetics II. Proceeding of 2nd International Barley Genetics Symposium* ed. Nilan R. A. (Pullman: Washington State University Press) 388–408

[B123] Tamaki S. Matsuo S. Wong H. L. Yokoi S. Shimamoto K. (2007). Hd3a protein is a mobile flowering signal in rice. *Science* 316 1033–1036 10.1126/science.1141753 17446351

[B124] Thines B. C Youn Y. Duarte M. I. Harmon F. G. (2014). The time of day effects of warm temperature on flowering time involve PIF4 and PIF5. *J. Exp. Bot.* 65 1141–1151 10.1093/jxb/ert487 24574484 PMC3935576

[B125] Thomashow M. F. (2010). Molecular basis of plant cold acclimation: insights gained from studying the CBF cold response pathway. *Plant Physiol.* 154 571–577 10.1104/pp.110.161794 20921187 PMC2948992

[B126] Trevaskis B. (2010). The central role of the VERNALIZATION1 gene in the vernalization response of cereals. *Funct. Plant Biol.* 37 479–487 10.1071/FP10056

[B127] Trevaskis B. Bagnall D. J. Ellis M. H. Peacock W. J. Dennis E. S. (2003). MADS box genes control vernalization-induced flowering in cereals. *Proc. Natl. Acad. Sci. U.S.A.* 100 13099–13104 10.1073/pnas.1635053100 14557548 PMC240751

[B128] Trevaskis B. Hemming M. N. Dennis E. S. Peacock W. J. (2007). The molecular basis of vernalization-induced flowering in cereals. *Trends Plant Sci.* 12 352–357 10.1016/j.tplants.2007.06.010 17629542

[B129] Trevaskis B. Hemming M. N. Peacock W. J. Dennis E. S. (2006). HvVRN2 responds to day length, whereas HvVRN1 is regulated by vernalization and developmental status. *Plant Physiol.* 140 1397–1405 10.1104/pp.105.073486 16500994 PMC1435809

[B130] Turck F. Fornara F. Coupland G. (2008). Regulation and identity of florigen: *FLOWERING LOCUS T* moves center stage. *Annu. Rev. Plant Biol.* 59 573–594 10.1146/annurev.arplant.59.032607.092755 18444908

[B131] Turner A. Beales J. Faure S. Dunford R. P. Laurie D. A. (2005). The pseudo-response regulator Ppd-H1 provides adaptation to photoperiod in barley. *Science* 310 1031–1034 10.1126/science.1117619 16284181

[B132] Turner A. S. Faure S. Zhang Y. Laurie D. A. (2013). The effect of day-neutral mutations in barley and wheat on the interaction between photoperiod and vernalization. *Theor. Appl. Genet.* 126 2267–2277 10.1007/s00122-013-2133-6 23737074 PMC3755224

[B133] Valverde F. Mouradov A. Soppe W. Ravenscroft D. Samach A. Coupland G. (2004). Photoreceptor regulation of CONSTANS protein in photoperiodic flowering. *Science* 303 1003–1006 10.1126/science.1091761 14963328

[B134] von Zitzewitz J. Szûcs P. Dubcovsky J. Yan L. L. Francia E. Pecchioni N. (2005). Molecular and structural characterization of barley vernalization genes. *Plant Mol. Biol.* 59 449–467 10.1007/s11103-005-0351-2 16235110

[B135] Watson L. Dallwitz M. J. (1992 onwards). *The Grass Genera of the World: Descriptions, Illustrations, Identification, and Information Retrieval; Including Synonyms, Morphology, Anatomy, Physiology, Phytochemistry, Cytology, Classification, Pathogens, World and Local Distribution, and References.* Version: 5th February 2014. Available at:

[B136] Wenkel S. Turck F. Singer K. Gissot L. Le Gourrierec J. Samach A. (2006). CONSTANS and the CCAAT box binding complex share a functionally important domain and interact to regulate flowering of *Arabidopsis*. *Plant Cell* 18 2971–2984 10.1105/tpc.106.043299 17138697 PMC1693937

[B137] Wigge P. A. Kim M. C. Jaeger K. E. Busch W. Schmid M. Lohmann J. U. (2005). Integration of spatial and temporal information during floral induction in *Arabidopsis*. *Science* 309 1056–1059 10.1126/science.1114358 16099980

[B138] Wilhelm E. P. Turner A. S. Laurie D. A. (2009). Photoperiod insensitive Ppd-A1a mutations in tetraploid wheat (*Triticum durum* Desf.). *Theor. Appl. Genet.* 118 285–294 10.1007/s00122-008-0898-9 18839130

[B139] Wu L. Liu D. Wu J. Zhang R. Qin Z. Liu D. (2013). Regulation of *FLOWERING LOCUS T* by a microRNA in *Brachypodium distachyon*. *Plant Cell* 25 4363–4377 10.1105/tpc.113.118620 24285787 PMC3875723

[B140] Wu Z. Q. Ge S. (2012). The phylogeny of the BEP clade in grasses revisited: evidence from the whole-genome sequences of chloroplasts. *Mol. Phylogenet. Evol.* 62 573–578 10.1016/j.ympev.2011.10.019 22093967

[B141] Xue W. Xing Y. Weng X. Zhao Y. Tang W. Wang L. (2008). Natural variation in Ghd7 is an important regulator of heading date and yield potential in rice. *Nat. Genet.* 40 761–767 10.1038/ng.143 18454147

[B142] Yan L. Fu D. Li C. Blechl A. Tranquilli G. Bonafede M. (2006). The wheat and barley vernalization gene VRN3 is an orthologue of FT. *Proc. Natl. Acad. Sci. U.S.A.* 103 19581–19586 10.1073/pnas.0607142103 17158798 PMC1748268

[B143] Yan L. Loukoianov A. Blechl A. Tranquilli G. Ramakrishna W. SanMiguel P. (2004a). The wheat VRN2 gene is a flowering repressor down-regulated by vernalization. *Science* 303 1640–1644 10.1126/science.1094305 15016992 PMC4737501

[B144] Yan L. Helguera M. Kato K. Fukuyama S. Sherman J. Dubcovsky J. (2004b). Allelic variation at the VRN-1 promoter region in polyploid wheat. *Theor. Appl. Genet.* 109 1677–1686 10.1007/s00122-004-1796-4 15480533

[B145] Yan L. Loukoianov A. Tranquilli G. Helguera M. Fahima T. Dubcovsky J. (2003). Positional cloning of the wheat vernalization gene VRN1. *Proc. Natl. Acad. Sci. U.S.A.* 100 6263–6268 10.1073/pnas.0937399100 12730378 PMC156360

[B146] Yang S. Weers B. D. Morishige D. T. Mullet J. E. (2014). CONSTANS is a photoperiod regulated activator of flowering in sorghum. *BMC Plant Biol.* 14:148 10.1186/1471-2229-14-148 PMC4046011 24884377

[B147] Zakhrabekova S. Gough S. P. Braumann I. Müller A. H. Lundqvist J. Ahmann K. (2012). Induced mutations in circadian clock regulator Mat-a facilitated short-season adaptation and range extension in cultivated barley. *Proc. Natl. Acad. Sci. U.S.A.* 109 4326–4331 10.1073/pnas.1113009109 22371569 PMC3306670

[B148] Zhang J. Wang Y. Wu S. Yang J. Liu H. Zhou Y. (2012). A single nucleotide polymorphism at the Vrn-D1 promoter region in common wheat is associated with vernalization response. *Theor. Appl. Genet.* 125 1697–1704 10.1007/s00122-012-1946-z 22875177

[B149] Zhao L. Zhang N. Ma P. F. Liu Q. Li D. Z. Guo Z. H. (2013). Phylogenomic analyses of nuclear genes reveal the evolutionary relationships within the BEP clade and the evidence of positive selection in Poaceae. *PLoS ONE* 8:e64642 10.1371/journal.pone.0064642 PMC3667173 23734211

[B150] Zhu J. Pearce S. Burke A. See D. R. Skinner D. Z. Dubcovsky J. (2014). Copy number and haplotype variation at the VRN-A1 and central FR-A2 loci are associated with frost tolerance in hexaploid wheat. *Theor. Appl. Genet.* 127 1183–1197 10.1007/s00122-014-2290-2 24626953 PMC4876961

